# The relationship between quality of life and physical fitness in people with severe mental illness

**DOI:** 10.1186/s12955-018-0909-8

**Published:** 2018-05-02

**Authors:** D. Perez-Cruzado, A. I. Cuesta-Vargas, E. Vera-Garcia, F. Mayoral-Cleries

**Affiliations:** 1 Department of Physiotherapy, Faculty of Health Sciences, University of Malaga, Av/ Arquitecto Peñalosa s/n, Malaga, Spain; 2 Clinimetric Research Group of Biomedicine Research Institute of Malaga (IBIMA) Spain, Regional University Hospital, Málaga, Spain; 3 School of Clinical Science, Faculty of Health Science, Queensland University Technology, Brisbane, QLD Australia; 4 Mental Health Research Group of Biomedicine Research Institute of Malaga (IBIMA) Spain, Regional University Hospital, Malaga, Spain

**Keywords:** Quality of life, Physical activity, Physical fitness, Fitness condition, Severe mental illness

## Abstract

**Background:**

Quality of life of people with severe mental illness may be decrease by the high occurrence of metabolic and cardiovascular diseases. Physical fitness emerges as a modifying factor in this population through physical activity and this modification could influence in the quality of life of this population. The aim of the present study is to determine the contribution of physical fitness to the quality of life of people with severe mental illness.

**Methods:**

In the current study, a physiotherapist and an occupational therapist assessed 62 people with severe mental illness. Physical fitness was measured with a range of 11 fitness tests that covered flexibility, strength, balance, and endurance. To assess quality of life the EQ-5D-3 L scale was used, which measures five dimensions (mobility, self-care, usual activities, pain-discomfort, and anxiety-depression).

**Results:**

Significant correlations are presented between the quality of life and primary variables of physical fitness (balance, endurance, and upper limb strength). Endurance explained 22.9% of the variance of the quality of life in people with severe mental illness. Functional reach added another 36.2% variance to the prediction of quality of life.

**Conclusions:**

The results of the present study suggest that some variables of physical fitness are associated with quality of life in people with severe mental illness. The improvement in physical fitness of this population should be a primary objective.

**Trial registration:**

ClinicalTrials.gov Identifier: NCT02413164 “retrospective registered” Registered Febr 2017.

## Background

Severe mental illness (SMI) includes a set of pathologies with, an ICD-10 diagnosis of an affective or non-affective functional psychotic disorder (codes F20-F22, F24, F25, F28-F31, F32.3, F33.3) [15]. People with SMI have consistently higher levels of mortality and morbidity than the general population [10, 16].Although suicide contributes to the increased mortality associated with schizophrenia, individuals with schizophrenia have increased mortality risks attributable to a wide range of comorbid somatic conditions and unhealthy lifestyles [26]. Many physical disorders have been identified that are more prevalent in individuals with SMI. It is a fact that patients with schizophrenia suffer:

1. A greater prevalence than the general population of the following: infections of hepatitis C and Human immunodeficiency virus (HIV), with little knowledge and concern about AIDS on the part of the patient; metabolic disorders (diabetes, glucose intolerance and metabolic syndrome or MetS), independent of their pharmacological treatments; spontaneous dyskinesia, even without antipsychotic treatment; respiratory disorders related to cigarette smoking (asthma, COPD and emphysema), and cardiovascular disorders.
2. Excessive overall mortality from natural causes: respiratory, digestive, genitourinary, cardiovascular, infectious, mental, and endocrine diseases [2, 17].

Given the potential for an even greater disease burden as a result of the introduction of second-generation antipsychotic medications, research aimed at optimising the physical health of people with schizophrenia needs to be undertaken with a sense of urgency in order to minimize the consumption of this type of medication [22, 25].

In people with schizophrenia or schizoaffective disorder, physical fitness seems to emerge as a major modifiable risk factor for obesity and metabolic and cardiovascular diseases, and overall morbidity and mortality [23, 27, 29].

In addition to modifiable lifestyle factors and psychotropic medication side effects, poorer access to, and quality of, received health care remain addressable problems for patients with SMI [30].

Health related quality of life is a measure of the impact of an illness and consequent upon functional health status and well-being as perceived by the patient [21]. The quality of life criterion is a valid and useful outcome in patients with schizophrenia. However, a lack of consensus on the scales hinders research into its predictive capacity [9]. Physical activity reduces symptoms of schizophrenia and improves anthropometric measures, aerobic capacity, and quality of life among people with mental illness [14].

The objective of the present study is to identify the relationship between the quality of life and a modifiable factor, such as the physical condition, in people with SMI. In this way, specific interventions could be undertaken to improve the levels of physical status and thereby modify the quality of life in this population.

## Methods

### Participants

The study procedure was approved by the Ethical Committee of Research of Málaga Northeast. Patients of the Mental Health Service of the Regional University Hospital of Málaga were recruited in November 2016, over a 6-month period. All subjects should have been admitted for more than 6 months and not be planned for discharge in the next 3 months. The sample included 62 participants (37 men and 25 women), between the ages of 26 and 61 years, diagnosed with SMI by the psychiatrists responsible for the patients’ treatment, using ICD-10. The sample was recruited by a researcher belonging to the research group. Before participating in the study, the purpose was explained to the participants, as well as their relatives, and the patients signed a written informed consent. Two patients were excluded, due to a comorbid diagnosis of substance abuse or a somatic problem, like cardiovascular, metabolic or musculoskeletal disorders, which would not allow us to perform the physical tests safely. None of the patients who were invited to participate in the study rejected this invitation.

### Study design and procedures

A cross-sectional study was performed. The sociodemographic variables were collected in a semi-structured interview. The anthropometrics measurements (body weight, height and waist circumference) were collected according to standards of the International Society for the Advancement of Kinanthropometry [20].

The assessors were a physiotherapist and an occupational therapist, who had previously been trained in the proper performance of the 11 physical tests that were assessed and EQ-5D-3 L scale. The scale was explained to the patients and the examiners demonstrated how to perform the tests.

### Outcome measures

A range of 11 physical tests were used to measure physical fitness: passive knee extension test, calf muscle flexibility test, anterior hip flexibility test, functional shoulder rotation test, timed-stand test, partial sit-up test, seated push-up test, grip test, single leg stance, open and closed eyes; functional reach test, and the two minute step test [3]. Those tests include measures of the different categories of physical condition (flexibility, balance, strength, and endurance).

To measure quality of life, the EQ-5D-3 L scale was employed. The EQ-5D-3 L assesses five dimensions: mobility, self-care, usual activities, pain-discomfort, and anxiety-depression. The respondents are asked to indicate their health state in each of the five dimensions according to one of three levels: “no problems”, “moderate problems”, or “severe problems” [1]. The second part of the EQ-5D-3 L is a visual analogue scale (VAS), a vertical 20 cm scale, ranging from zero (worst imaginable health state) to 100 (best imaginable health state). The individuals must mark the point on the vertical line that best reflects their assessment of their global health status as it is at this point. The use of the VAS provides a complementary score to the descriptive system of self-assessment of the health status of the individual [8]. All tests and the scale were measured on the same day. Each item of the EQ-5D-EL scale was explained if the participant could not understand or if a mistake could occur. Conversely, physical fitness tests were described repeatedly and physically demonstrated to ease the anxiety of the participants. Participants could attempt the exercises before the measurement. Once the analysis was completed, all participants received information about their physical profile and recommendations on how to increase their physical qualities.

### Data analysis

Descriptive data for all variables were presented as mean and standard deviation. To check the homogeneity of the sample, the Kolmogorov-Smirnov test was used to decide whether a parametric or a nonparametric statistic would be used. A *P-value* < 0.05 was considered to be statistically significant [4].

A correlation between .30 and .60 was considered to be mild and stronger correlations were considered to be high [12].

A linear regression analysis was performed to assess the independent variables with EQ-5D-3 L scale, after control for covariates (age and gender) to know what the physical condition contributes to the general quality of life of people with severe mental illness. Data analysis was performed with SPSS version 22.0 statistical software.

## Results

The mean age of the participants was 45.69 (± 8.04) years old. The mean weight was 81.26 (± 15.84) kilograms, and mean height was 165 (± 20.81) centimetres. The body mass index was 30.63 (± 5.24). The mean and standard deviation of quality of life scale and physical tests are presented in Table 1.

**Table 1:** Characteristics of participants

		Men	Women
	Cutoffs	Mean (sd)	Mean (sd)
Age		43.70 (7.86)	47.68 (8.22)
Height (cm)		169.62 (22.28)	159.92 (19.34)
Wheight (kg)		89.21 (18.30)	73.29 (13.38)
BMI		30.24 (5.01)	31.02 (5.47)
Waist circunference		105.02 (14.07)	100.88 (12.99)
Cigarette/day		26.62 (12.29)	17.96 (8.65)
QoL	0–1	0.63 (0.20)	0.67 (0.22)
VAS	0–100	64.76 (18.44)	68.46 (18.88)
PKE_R(°)	(−30−30)	−26.46 (14.95)	−21.22 (13.45)
PKE_L(°)	(−30−30)	−18.98 (13.26)	−20.36 (13.94)
CMF_R(°)	(−15−0)	−0.87 (3.24)	−0.95 (3.02)
CMF_L(°)	(−15−0)	−1.83 (5.55)	− 1.91 (5.29)
AHF_R(°)	(−10−0)	−0.31 (1.87)	−0.33 (1.69)
AHF_L(°)	(−10−0)	−0.30 (1.88)	−0.34 (1.78)
FRS_R(cm)	(−21−14)	−10.83 (12.34)	−11.01 (12.12)
FRS_L(cm)	(−21−14)	−16.85 (14.05)	−16.55 (13.87)
SPU(s)	(0−60)	30.00 (15.46)	28.34 (16.12)
PSUT(max. repetition/1 m)	(0−45)	30.07 (15.03)	24.39 (12.75)
TST(s)	(5−60)	17.96 (9.61)	12.56 (8.63)
HGT(max. kg)	(0−47)	32.14 (14.02)	23.06 (12.14)
SLSEO(s)	(0−30)	15.50 (10.67)	14.22 (10.01)
SLSEC(s)	(0−30)	6.93 (6.59)	7.01 (6.67)
FRT(cm)	(0−60)	35.94 (9.16)	35.28 (9.48)
2MEST_BE(bpm)	(60−110)	88.64 (16.99)	87.88 (16.03)
2MEST_AE(bpm)	(80−140)	106.14 (20.26)	116.34 (22.32)
2MEST_2MA(bpm)	(60−110)	89.30 (15.93)	93.82 (17.03)

*N* = 62

*BMI* Body mass index, *QoL* Quality of life of EQ-5D-3 L Scale, *VAS* Visual analogue scale of EQ-5D-3 L Scale, *PKE_R* Right passive knee extension, *PKE_L* Left passive knee extension, *CMF_R* Right calf muscle flexibility, *CMF_L* Left calf muscle flexibility, *AHF_R* Right anterior hip flexibility, *AHF_L* Left anterior hip flexibility, *FSR_R* Right functional shoulder rotation, *FSR_L* Left functional shoulder rotation, *SPU* Seated push-up, *PSUT* Partial sit-up test, *TST* Time-stands test, *HGT* Handgrip test, *SLSEO* Single-leg stance with opened eyes, *SLSEC* Single-leg stance with closed eyes, *FRT* Functional reach test, *2MEST_BE* Two-minute step test_before exercise, *2MEST_AE* Two-minute step test_before exercise, *2MEST_2MA* Two-minute step test_2 minute after

Table 2 shows the correlations between the EQ-5D-3 L scale and physical tests. Significant correlations were found between quality of life and balance (*r* = 0.360) and endurance (*r* = 0.312). It is important to highlight the significant correlation found between upper limb strength and the VAS of quality of life (*r* = 0.306).

**Table 2:** Correlations (Pearson r) between quality of life and physical tests

		QoL	VAS
Flexibility	PKE_R	.05 (.06)	.01 (.07)
	PKE_L	−.02 (.11)	−.03 (.17)
	CMF_R	.06 (.13)	.12 (.06)
	CMF_L	−.12 (.07)	.17 (.09)
	AHF_R	.06 (.06)	.08 (.14)
	AHF_L	.06 (.17)	.08 (.09)
	FRS_R	−.07 (.14)	.10 (.15)
	FRS_L	−.09 (.18)	.09 (.17)
Strength	SPU	.15 (.08)	.31* (.01)
	PSUT	−.20 (.06)	−.06 (.15)
	TST	−.33 (.06)	−.16 (.22)
	HGT	.12 (.11)	.10 (.09)
Balance	SLSEO	.14 (.22)	.13 (.18)
	SLSEC	.10 (.09)	.02 (.13)
	FRT	.36*(.02)	.25 (.08)
Endurance	2MEST_BE	.10 (.21)	.01 (.10)
	2MEST_AE	.31*(.02)	.02 (.07)
	2MEST_2MA	−.17 (.11)	.06 (.13)

*QoL* Quality of life of EQ-5D-3 L Scale, *VAS* Visual analogue scale of EQ-5D-3 L Scale, *PKE_R* Right passive knee extension, *PKE_L* Left passive knee extension, *CMF_R* Right calf muscle flexibility, *CMF_L* Left calf muscle flexibility, *AHF_R* Right anterior hip flexibility, *AHF_L* Left anterior hip flexibility, *FSR_R* Right functional shoulder rotation, *FSR_L* Left functional shoulder rotation, *SPU* Seated push-up, *PSUT* Partial sit-up test, *TST* Time-stands test, *HGT* Handgrip test, *SLSEO* Single-leg stance with opened eyes, *SLSEC* Single-leg stance with closed eyes, *FRT* Functional reach test, *2MEST_BE* Two-minute step test_before exercise, *2MEST_AE* Two-minute step test_before exercise, *2MEST_2MA* Two-minute step test_2 minute after

*p < 0,05

The regression analyses with EQ-5D-3 L scale showed that the variance in quality of life for physical fitness could be explained 49% (Model Summary *r*^*2*^ = 0.26; F 4.34; *p* = 0.01) with a level of contribution of each predictor of β =0.23 by endurance and in β =0.36 by the test ‘functional reach’ (Table 3). A regression analyses with EQ5D-3 L was performed with demographic data (Age) and Functional Reach test (Model Summary *r*^*2*^ = 0.13; F 3.18; *p* = 0.05), with a level of contribution of β =0.22 by Functional Reach test, in contrast, the level of contribution of demographic data (Age) was not significant (Table 4).

**Table 3:** Regression analyses with EQ-5D-3 L scale scores as the dependent variables (Endurance and Functional Reach test)

Variables^a^	B	SE	β	t	p
Endurance	0.01	0.01	0.23	1.04	0.05
Functional Reach test	0.01	0.01	0.36	2.82	< 0.01

^a^Only significant correlates were included in the model; *B* unstandardized coefficient, *SE* standard error, *β* standardized coefficient

**Table 4:** Regression analyses with EQ-5D-3 L scale scores as the dependent variables (Functional Reach test and Age)

Variables	B	SE	β	t	p
Functional Reach test	0.01	0.01	0.22	1.73	0.05
Age	0.01	0.01	−0.18	−1.47	0.08

*B* unstandardized coefficient, *SE* standard error, *β* standardized coefficient

## Discussion

The current study was developed to determine the relationship between physical fitness and quality of life in people with SMI.

Positive mild correlations were found between a few variables of quality of life and strength, balance and endurance. This was the first study that related different primary physical variables (flexibility, strength, balance and endurance) within the concept of physical fitness to quality of life in people with SMI, but endurance and quality of life were related in people with schizophrenia in an previous study [24].

The study of Vancampfort et al. [24, 29] assessed endurance in people with schizophrenia by the Astrand–Rhyming cycle ergometer test and the quality of life through the 36-item Short-Form Health Survey (SF-36). Despite using different scales to assess these variables, Vancampfort et al. [24, 29] found a significant correlation between quality of life (physical component) (*r* = 0.57; *p* < 0.01), similarly to the results found in the current study (*r* = 0.31; *p* < 0.05). This finding is clinically relevant, due to the importance of aerobic condition, regarding the risk of death in people with SMI [31]. Nonetheless, the present manuscript adds further information about the association between physical fitness and quality of life in people with severe mental illness, by considering flexibility, strength and balance.

The present study gives us more information about the relationship between physical fitness and quality of life: variables were included that had not been previously described as factors relevant to quality of life in people with SMI. The current research found significant correlations between quality of life and balance (*r* = 0.36; *p* < 0.05) and strength (*r* = 0.31; *p* < 0.05). These could be relevant factors to the quality of life of this population.

There are reasons to hypothesise about the relationship of these variables with quality of life. As Vancampfort et al. [24, 29] states, aerobic condition improves the capacity of muscles without the appearance of fatigue; as a result there is an improvement in the independence in activities of daily life [11]. Moreover, aerobic condition plays a key role in people with SMI; a worse aerobic condition is related to a higher risk of death in people with SMI [31]. On the other hand, muscular strength and balance are important variables in quality of life, as they are paramount in healthy ageing, improving independence in the activities of daily life, and decreasing the risk of falls [5, 11]. As shown in the backward stepwise regression analysis, the quality of life in people with SMI could be explained by dynamic balance in 26.3% and by endurance in 22.9%, with almost 50% of the variance of the overall quality of life in this population.

The present findings must be interpreted with caution because of some methodological limitations. First, the study was cross-sectional and, therefore, we cannot establish cause and effect. However, it has been demonstrated that the use of longitudinal studies in HRQOL research is also pivotal, to control for unobserved heterogeneity [7]. Second, the sample size was rather small and only included participants limited to a single centre, which reduces the generalisability.

Another limitation is the relatively limited number of variables examined; in addition to sociodemographic and clinical data, a complex interaction of other factors, such as self-esteem, premorbid adjustment, therapy, and social support network could also play a role in determining subjective quality of life.

Finally, a potential limitation of the study was the use of the EQ-5D-3 L, a generic questionnaire that may not have detected subtle changes in subjective quality of life in the specific population of schizophrenia patients. However, the huge variation in quality of life scales used in patients with schizophrenia complicates the comparison of results of different studies. Future research needs to find more consensus on the concept and measures of quality of life.

Although there are these limitations, the data shown in the current study have important practice implications. In spite of lower levels of quality of life found in people with SMI and the difficulty in achieving recovery, the data in the current study suggest that some variables of physical fitness may be associated with quality of life in people with severe mental illness.

Physical activity should be encouraged in people with SMI as it is an important factor in improving physical fitness, and physical activity is closely related to the quality of life of this population ([6]; Andy [18, 19, 28]). Nonetheless it is important to develop interventions with the aim of removing the barriers that people with SMI find when participating in physical activity focused on the improvement of the endurance and balance ([13]; Andrew [19]).

## Conclusion

The current study shows how physical fitness (strength, flexibility and balance) may be a contributing factor to the quality of life in people with SMI. Thus, it highlights the importance of the physical fitness since the quality of life of people with severe mental illness may be influence by this. This study is the first to show the relationship between physical status and quality of life in people with SMI. Accordingly, it would be beneficial to encourage programs focused on improving the physical health of this population, which can influence their quality of life, overall.

## Abbreviations

HIB: Human immunodeficiency virus
MET: Metabolic equivalent of task
SMI: Severe mental illness
SPSS: Statistical analysis software package
VAS: Visual analogue scale

## References

[1] Badia X, Roset M, Montserrat S, Herdman M, Segura A (1999). The Spanish version of EuroQol: a description and its applications. European Quality of Life scale. Med Clín.

[2] Bobes-García J, Saiz-Ruiz J, Bernardo-Arroyo M, Caballero-Martínez F, Gilaberte-Asín I, Ciudad-Herrera A (2012). Delphi consensus on the physical health of patients with schizophrenia: evaluation of the recommendations of the Spanish societies of psychiatry and biological psychiatry by a panel of experts. Actas Esp Psiquiatr.

[3] Cuesta-Vargas AI, Paz-Lourido B, Rodriguez A (2011). Physical fitness profile in adults with intellectual disabilities: differences between levels of sport practice. Res Dev Disabil.

[4] Fisher RA, Yates F, Fisher RA, Yates F (1963). Statistical tables for biological, agricultural and medical research.

[5] García-Flores FI, Rivera-Cisneros AE, Sánchez-González JM, Guardado-Mendoza R, Torres-Gutiérrez JL. Correlation between gait speed and muscular strength with balance for reducing falls among elderly. Cir Cir. 2016; 10.1016/j.circir.2015.12.005 10.1016/j.circir.2015.12.005 26945638

[6] Gomes E, Bastos T, Probst M, Ribeiro JC, Silva G, Corredeira R. Quality of life and physical activity levels in outpatients with schizophrenia. Rev Bras Psiquiatr (Sao Paulo, Brazil: 1999). 2016; 10.1590/1516-4446-2015-1709 10.1590/1516-4446-2015-1709 PMC7111367 26814836

[7] Hajek A, Brettschneider C, Mallon T, Ernst A, Mamone S, Wiese B et al. The impact of social engagement on health-related quality of life and depressive symptoms in old age-evidence from a multicenter prospective cohort study in Germany. Health Qual Life Outcomes. 2017;14;15(1):140. 10.1186/s12955-017-0715-8 PMC5513118 28705225

[8] Herdman M, Badia X, Berra S (2001). El EuroQol-5D: una alternativa sencilla para la medición de la calidad de vida relacionada con la salud en atención primaria. Atención Primaria.

[9] Karow A, Wittmann L, Schöttle D, Schäfer I, Lambert M (2014). The assessment of quality of life in clinical practice in patients with schizophrenia. Dialogues Clin Neurosci.

[10] McGrath J, Saha S, Chant D, Welham J (2008). Schizophrenia: a concise overview of incidence, prevalence, and mortality. Epidemiol Rev.

[11] Pate RR (1988). The evolving definition of physical fitness. Quest.

[12] Portney LG, Watkins MP (2009). Foundations of clinical research: applications to practice.

[13] Rastad C, Martin C, Asenlöf P (2014). Barriers, benefits, and strategies for physical activity in patients with schizophrenia. Phys Ther.

[14] Rosenbaum S, Tiedemann A, Sherrington C, Curtis J, Ward PB (2014). Physical activity interventions for people with mental illness: a systematic review and meta-analysis. J Clin Psychiatry.

[15] Ruggeri M, Leese M, Thornicroft G, Bisoffi G, Tansella M (2000). Definition and prevalence of severe and persistent mental illness. Br J Psychiatry J Ment Sci.

[16] Saha S, Whiteford H, McGrath J (2014). Modelling the incidence and mortality of psychotic disorders: data from the second Australian national survey of psychosis. Aust N Z J Psychiatry.

[17] Sáiz Ruiz J, Bobes García J, Vallejo Ruiloba J, Giner Ubago J, García-Portilla González MP, Grupo de Trabajo sobre la Salud Física del Paciente con Esquizofrenia (2008). Consensus on physical health of patients with schizophrenia from the Spanish societies of psychiatry and biological psychiatry. Actas Esp Psiquiatr.

[18] Soundy A, Freeman P, Stubbs B, Probst M, Coffee P, Vancampfort D (2014). The transcending benefits of physical activity for individuals with schizophrenia: a systematic review and meta-ethnography. Psychiatry Res.

[19] Soundy A, Stubbs B, Probst M, Hemmings L, Vancampfort D (2014). Barriers to and facilitators of physical activity among persons with schizophrenia: a survey of physical therapists. Psychiatr Serv (Washington, D.C.).

[20] Stewart AA, Marfell-Jones M, Olds T, Al E (2011). International standards for anthropometric assessment.

[21] van Os J, Kapur S (2009). Schizophrenia. Lancet (London, England).

[22] Vancampfort D, De Hert M, Skjerven LH, Gyllensten AL, Parker A, Mulders N (2012). International Organization of Physical Therapy in mental health consensus on physical activity within multidisciplinary rehabilitation programmes for minimising cardio-metabolic risk in patients with schizophrenia. Disabil Rehabil.

[23] Vancampfort D, De Hert M, Sweers K, De Herdt A, Detraux J, Probst M (2013). Diabetes, physical activity participation and exercise capacity in patients with schizophrenia. Psychiatry Clin Neurosci.

[24] Vancampfort D, Guelinckx H, Probst M, Stubbs B, Rosenbaum S, Ward PB, De Hert M (2015). Health-related quality of life and aerobic fitness in people with schizophrenia. Int J Ment Health Nurs.

[25] Vancampfort D, Probst M, Helvik Skjaerven L, Catalán-Matamoros D, Lundvik-Gyllensten A, Gómez-Conesa A (2012). Systematic review of the benefits of physical therapy within a multidisciplinary care approach for people with schizophrenia. Phys Ther.

[26] Vancampfort D, Probst M, Knapen J, Carraro A, De Hert M (2012). Associations between sedentary behaviour and metabolic parameters in patients with schizophrenia. Psychiatry Res.

[27] Vancampfort D, Probst M, Scheewe T, De Herdt A, Sweers K, Knapen J (2013). Relationships between physical fitness, physical activity, smoking and metabolic and mental health parameters in people with schizophrenia. Psychiatry Res.

[28] Vancampfort D, Probst M, Scheewe T, Maurissen K, Sweers K, Knapen J, De Hert M (2011). Lack of physical activity during leisure time contributes to an impaired health related quality of life in patients with schizophrenia. Schizophr Res.

[29] Vancampfort D, Wyckaert S, Sienaert P, De Hert M, Stubbs B, Buys R (2015). The functional exercise capacity in patients with bipolar disorder versus healthy controls: a pilot study. Psychiatry Res.

[30] Wang X-Q, Petrini M, Morisky DE (2016). Comparison of the quality of life, perceived stigma and medication adherence of Chinese with schizophrenia: a follow-up study. Arch Psychiatr Nurs.

[31] Wildgust HJ, Hodgson R, Beary M (2010). The paradox of premature mortality in schizophrenia: new research questions. J Psychopharmacol (Oxford, England).

